# Target-dependent effects of rTMS on social disability in patients with obsessive-compulsive disorder

**DOI:** 10.1192/j.eurpsy.2026.10249

**Published:** 2026-07-31

**Authors:** G. D’Urso, B. Benatti, F. Perrotta, M. Manzo, M. V. Pomes, R. Rossi, M. Vismara, B. Dell’Osso

**Affiliations:** 1 Department of Psychiatry, University of Campania “Luigi Vanvitelli”, Naples; 2 University of Milan, ASST Fatebenefratelli - Sacco, Milan; 3 Department of neurosciences, reproductive and odontostomatological sciences, University of Naples Federico II, Naples; 4 Department of Mental Health and Addiction, Psychiatric Service of Diagnosis and Care - ASL Rome 5, Tivoli; 5 Department of Systems’ Medicine, University of Rome Tor Vergata, Rome, Italy

## Abstract

**Introduction:**

Obsessive-compulsive disorder (OCD) is a persistent and severely impairing mental health condition. Repetitive transcranial magnetic stimulation (rTMS) has emerged as a promising option for patients unresponsive to standard treatments. However, the optimal cortical target for maximizing clinical outcomes remains unclear.

**Objectives:**

This study compared the effects of low-frequency rTMS (LF-rTMS) over two different cortical targets for reducing OCD symptom severity and associated functional impairment.

**Methods:**

Thirty patients with treatment-resistant OCD were consecutively enrolled in two OCD clinics and assigned to receive twenty sessions of 1 Hz 30min rTMS over either right orbitofrontal cortex (R-OFC) or bilateral pre-supplementary motor area (BL-preSMA). Twelve patients in the R-OFC group and fifteen in the BL-preSMA group completed the treatment. Clinical evaluations included the Yale-Brown Obsessive-Compulsive Scale (Y-BOCS) for OCD symptom severity and the Sheehan Disability Scale (SDS) for functional impairment, assessed at baseline (T0), after one week (T1), two weeks (T2), and at the end of treatment (T3). Outcomes were analyzed using Linear Mixed Models (LMM).

**Results:**

Both protocols produced significant OCD symptom reductions from baseline to T1 (Coefficient = -4.54, p = 0.008) and T3 (Coefficient = -5.64, p = 0.013), with no differences between groups. A significant treatment × time interaction emerged for SDS scores. R-OFC stimulation led to functional improvement at T2 (Coefficient = -4.94, p = 0.034) and T3 (Coefficient = -5.27, p = 0.05), while BL-preSMA stimulation was associated with relative worsening at T1 (Coefficient = 8.29, p = 0.001), T2 (Coefficient = 10.54, p < 0.001), and T3 (Coefficient = 9.27, p = 0.011). Higher baseline disability predicted greater symptom reduction in BL-preSMA patients at T2 (Coefficient = 0.75, p = 0.022) and T3 (Coefficient = 1.18, p = 0.005).

**Conclusions:**

R-OFC and BL-preSMA rTMS treatment both produced OCD symptom severity reduction, while R-OFC stimulation was more effective in reducing disability, and BL-preSMA stimulation was more beneficial for patients with higher baseline functional impairment. These results highlight the importance of personalizing rTMS treatment of OCD patients based also on their associated disability.

**Disclosure of Interest:**

None Declared

